# Cytotoxicity Evaluation of Chloroquine and Hydroxychloroquine in Multiple Cell Lines and Tissues by Dynamic Imaging System and Physiologically Based Pharmacokinetic Model

**DOI:** 10.3389/fphar.2020.574720

**Published:** 2020-11-20

**Authors:** Jianling Yang, Zhengyang Guo, Xu Liu, Qi Liu, Meng Wu, Xueting Yao, Yang Liu, Cheng Cui, Haiyan Li, Chunli Song, Dongyang Liu, Lixiang Xue

**Affiliations:** ^1^ Center of Basic Medicine Research (CBMR), Peking University Third Hospital, Beijing, China; ^2^ Drug Clinical Trial Center, Peking University Third Hospital, Beijing, China; ^3^ Department of Orthopedics, Peking University Third Hospital, Beijing, China; ^4^ Savaid Medical School, University of Chinese Academy of Sciences, Beijing, China

**Keywords:** ratio of tissue trough concentrations, dynamic imaging system, cytotoxicity, chloroquine and hydroxychloroquine, physiologically based pharmacokinetic model

## Abstract

Chloroquine (CQ) and hydroxychloroquine (HCQ) have been challenged in treating COVID-19 patients and still under debate due to the uncertainty regarding the effectiveness and safety, and there is still lack of the systematic study on the toxicity of these two drugs. To further uncover the toxicity profile of CQ and HCQ in different tissues, we evaluated the cytotoxicity of them in eight cell lines and further adopted the physiologically based pharmacokinetic models to predict the tissue risk, respectively. Retina, myocardium, lung, liver, kidney, vascular endothelium, and intestinal epithelium originated cells were included in the toxicity evaluation of CQ and HCQ, respectively. The proliferation pattern was monitored in 0–72 h by IncuCyte S3. CC50 and the ratio of tissue trough concentrations to CC50 (R_TTCC_) were brought into predicted toxicity profiles. Compared to CQ, HCQ was found to be less toxic in six cell types except Hep3B and Vero cells. In addition, R_TTCC_ was significantly higher in CQ treatment group compared to HCQ group, which indicates relative safety of HCQ. To further simulate the situation of the COVID-19 patients who suffered the dyspnea and hypoxemia, we also tested the cytotoxicity upon hypoxia and normoxia (1, 5 vs. 21% O_2_). It was found that the cytotoxicity of CQ was more sensitive to hypoxia compared with that of HCQ, particularly in liver originated cells. Both CQ and HCQ showed cytotoxicity in time-dependent manner which indicates the necessity of short period administration clinically.

## Introduction

The severe acute respiratory syndrome coronavirus 2 (SARS-CoV-2) has spread globally due to its high transmissibility and infectivity, resulting in an unprecedented global public health challenge (Rothe et al., 2020; Sohrabi et al., 2020). As of June 9, 2020, more than 7,000,000 cases have been confirmed around the world, according to data supplied by World Health Organization, and at least 400,000 people have died from the disease.

Treatments are urgently needed to cure respiratory failure and deaths caused by coronavirus disease 2019 (covid-19; Lu, 2020). Several *in vitro* studies have reported the activity by chloroquine (CQ) and hydroxychloroquine (HCQ) against severe acute respiratory syndrome coronavirus 2 (SARS-CoV-2) (Colson et al., 2020; Liu et al., 2020; Wang et al., 2020; Yao et al., 2020). Both CQ and HCQ have received worldwide attention as a choice for covid-19 in a short time. However, Borba MGS et al. have reported the first death case diagnosed as the cardiopathy by using CQ in Brazil, suggesting the nonnegligible side effect in the treatment for covid-19 patients (Borba et al., 2020). Besides the primary inflammation in the lungs and the reported cardiopathy, the autopsies also find that multiorgan failure and damage exist, particularly in the individuals with chronic underlying diseases or the elders (Bonow et al., 2020; Fihn et al., 2020; Geleris et al., 2020; Mahevas et al., 2020; Mercuro et al., 2020; Rothe et al., 2020). Therefore, the safety and effectiveness of CQ and HCQ in the treatment of new coronavirus pneumonia are still controversial, which need further study.

CQ belongs to the class of organic compounds known as 4-aminoquinolines, and HCQ is a derivative of CQ. These are organic compounds containing an amino group attached to the 4-position of a quinoline ring system (Supplementary Figure S1). CQ, together with its derivate HCQ, has been commercialized as antimalarial drugs in the clinic for several decades. HCQ has also been broadly used in autoimmune diseases treatment, such as systemic lupus erythematosus (SLE) and rheumatoid arthritis (Ezra and Jorizzo, 2012; Jallouli et al., 2013; Schultz et al., 2015; Concha et al., 2019). However, CQ and HCQ still have some potential concerns with prolonged usage, including heart rhythm disturbances, gastrointestinal upset, and retinal toxicity (Olansky, 1982; Savarino et al., 2003; Jallouli et al., 2013; Bahloul et al., 2017; Fernandez, 2017). Moreover, the toxicity of chloroquine and hydroxychloroquine in other tissues and organs was largely neglected. As the treatment of malaria and autoimmune diseases and candidate for other diseases, it is urgent to reevaluate and recognize the toxicity profile of chloroquine drugs in tissues and organs under certain circumstance, such as hypoxemia which is a very common symptom in COVID-19 pneumonia. This new study attempts to fill the gap in an area that has been neglected.

Toxicity tolerability in key tissues about drug effectiveness and side effect was critical to understand their mechanism and to optimize dosing regimen by integrating predicted tissue concentrations (TCs) of both drugs in patients. Therefore, comparison of tissue tolerable concentration and predicted concentration in each given tissue and cell line can be utilized to suggest dosing optimizing strategy for patients, especially in high risk populations. In current study, eight different types of cell lines including retina, myocardium, lung, liver, kidney, vascular endothelium, and intestinal epithelium originated cells were included in the cytotoxic evaluation with the presence of CQ or HCQ in 0–72 h on Incucyte S3, which could perform long-term continuous imaging and provide the cellular proliferation pattern upon drug treatment. Considering the potential impact on toxicity by low oxygen concentration, we further conducted the cytotoxic evaluation of CQ and HCQ by Incucyte S3 at 1% and 5% oxygen concentration. Consequently, the CC50 of CQ and HCQ combined with the predicted tissue concentration based on PBPK model was calculated at the given target organ, respectively. R_TTCC_ value, the ratio of simulated tissue trough concentration to CC50 (R_TTCC_), was introduced to predict the risk of tissue toxicity. The data suggest that HCQ was demonstrated to be much less toxic than CQ, at least at certain key tissues (heart, liver, kidney, and lung). Upon hypoxia, the cytotoxicity tends to increase upon HCQ treatment in HEK293 and H9C2 and CQ treatment in Hep3B and H9C2, which indicates it is more sensitive to oxygen concentration. Taken together, this study provides more detailed information regarding cytotoxicity in a wide spectrum and will be beneficial for both pharmacologists and physicians.

## Materials and Methods

### Compounds

Hydroxychloroquine sulphate of 99.7% purity was provided by Shanghai Zhongxi Sunve Pharmaceutical Co., Ltd., and chloroquine diphosphate of 96% purity was purchased from Beijing InnoChem Science & Technology Co., Ltd.

### Cell Culture

Vero, derived from kidney of *Cercopithecus aethiops*, and H9C2 cells, derived from heart tissue, were maintained in Dulbecco’s Modified Eagle’s Medium (DMEM) H-Glu (4.5 g/L Glucose). Human lung carcinomatous cell lines A549 cells were maintained in McCoy’s 5A Media (Modified with Tricine), hepatocellular carcinoma cells Hep3B and human embryonic kidney cell line HEK293 cells were maintained in Minimum Essential Medium (MEM Eagles with Earle’s Balanced Salts) (MEM-EBSS), and rat small intestinal epithelial cell line IEC-6 cells were cultured in DMEM supplemented with 0.01 mg/ml bovine insulin; these cell lines were acquired from National Infrastructure of Cell Line Resource. Human normal lung fibroblast cell line IMR-90 cells (ATCC CCL186) were maintained in MEM, 1% glutamine, 1% NEAA, and 1% sodium pyruvate. Human retinal pigment epithelia cell line ARPE-19 cells (Beiluo, CN) were maintained in DMEM/F12(1:1). All these cell lines were cultured with 10% (v/v) fetal bovine serum (Gibco-BRL, Invitrogen, Paisley, United Kingdom), penicillin, and streptomycin at 100 U/ml, at 37°C in a 5% CO_2_ incubator.

### Cell Proliferation Assay

To access the toxicity of chloroquine and hydroxychloroquine, these cells were treated by different concentration including 0.01, 0.03, 0.1, 0.3, 1, 3, 10, 30, 100, 300, and 1,000 μM, respectively. To detect the effects of drug metabolites, 50 μM CQ or HCQ was pretreated with S9 mix at 37°C for 3 h and centrifuged to collect the supernatant. Cells were seeded at a density of 8,000–10000 cells per well in a 96-well plate and maintained in regular medium for 72 h. Cell proliferation was monitored by a long-term process live cell analysis system IncuCyte S3 (Essen Instruments, Ann Arbor, MI, United States), where the cell proliferation was assessed by confluence measurements and normalized to 0 h calculated by IncuCyte software. Photographs of cells were taken at 3 h intervals from four separate regions per well with a 10× objective. Values from four regions of each well were pooled and averaged across three replicates.

### Physiologically Based Pharmacokinetic Model and Simulation

The PBPK models for CQ and HCQ were developed using Simcyp simulator (version 18). Simcyp Limited (a Certara company, Blades Enterprise Center, Sheffield, United Kingdom) provided CQ compound file and the HCQ compound file was self-built. Physical and chemical parameters were obtained from the literature. Pharmacokinetic parameters, such as liver intrinsic clearance, Fa, and Ka, were determined from clinical data and in vitro study. The lung to blood concentration ratio for CQ and HCQ (obtained from animal studies) was used to predict the drug concentration in the lungs, heart, liver, and kidney. Data obtained from the literature in graphical form were extracted using Plot Digitizer (version 2.26, GetData). Pharmacokinetic parameters that could not be sourced from the literature were estimated using extracted data in Phoenix (version 8.6, Certara company). The variation range of the calculated Rt value (the ratio of predicted to observed data) should be within 2-fold, namely, 0.5 ≤ Rt ≤ 2.0. Simulated HCQ and CQ dosage regimens were according to one clinical trial (Registration number: ChiCTR2000029899) and the “Diagnosis & Treatment Scheme for Novel Coronavirus Pneumonia (Trial) 6th Edition” enacted by the National Health Commission of the People’s Republic of China.

### Statistical Analysis

The two-tailed Student *t*-test was used to compare two groups of continuous variables. One-way ANOVA was used to determine the statistical signiﬁcance of three groups. A *p* value of less than 0.05 was considered statistically significant.

## Results

### The Effect of Chloroquine and Hydroxychloroquine on Cell Proliferation

**FIGURE 1:**
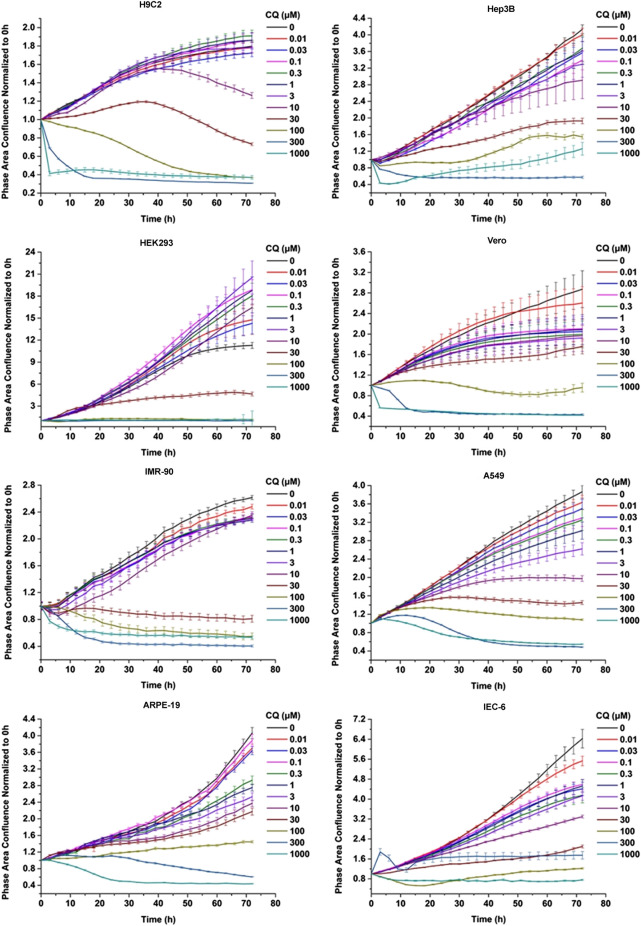
Chloroquine inhibited the viability of the eight cells in a dose- and time-dependent manner. CQ inhibited the viability of Vero cells and IMR-90, A549, H9C2, HEK293, Hep3B, and ARPE-19 cells in a dose- and time-dependent manner. These cells were seeded at a density of 3,000–5,000 cells per well in a 96-well plate and maintained in regular medium for 72 h, with different concentration of chloroquine including 0.01, 0.03, 0.1, 0.3, 1, 3, 10, 30, 100, 300, and 1,000 μM, respectively. The cell proliferation was assessed by confluence measurements normalized to 0 h calculated using IncuCyte (Essen BioScience). The experiments were performed in triplicate.

**FIGURE 2:**
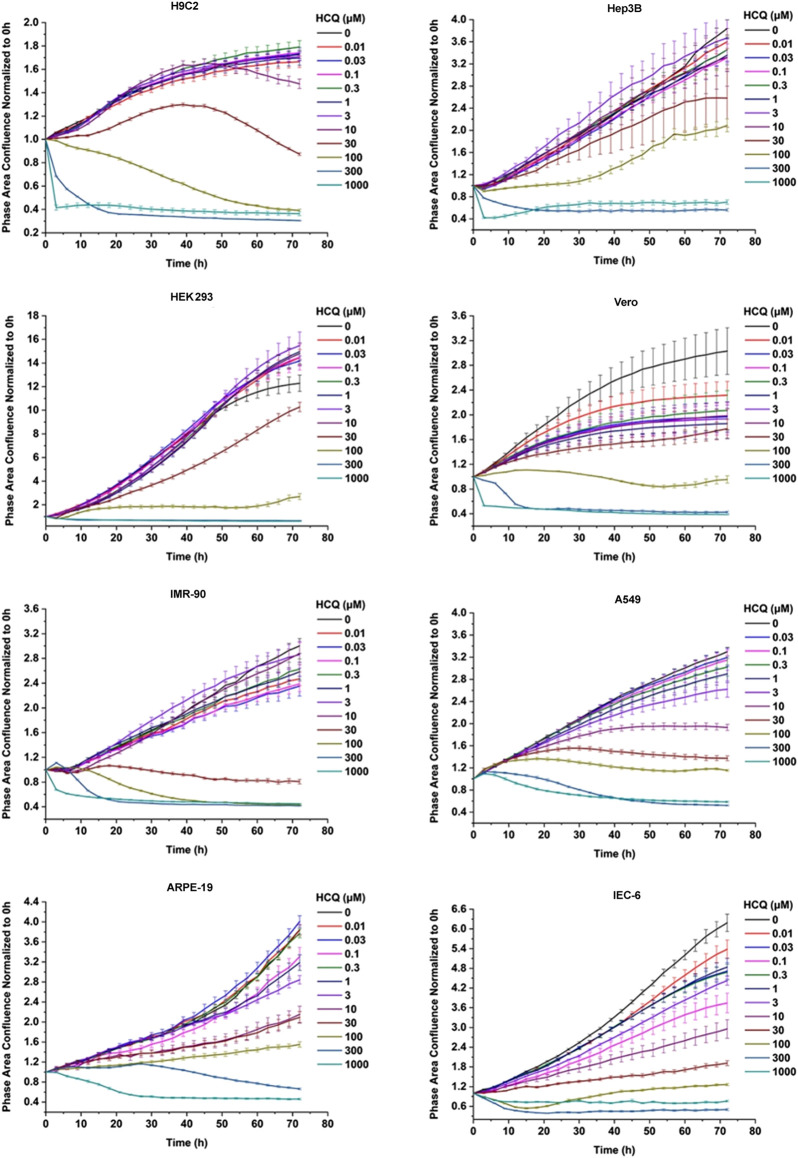
Hydroxychloroquine inhibited the viability of the eight cells in a dose- and time-dependent manner. HCQ inhibited the viability of Vero, IMR-90, A549, H9C2, HEK293, Hep3B, and ARPE-19 cells in a dose- and time-dependent manner. These cells were seeded at a density of 3,000–5,000 cells per well in a 96-well plate and maintained in regular medium for 72 h, with different concentration of Hydroxychloroquine including 0.01, 0.03, 0.1, 0.3, 1, 3, 10, 30, 100, 300, and 1,000 μM, respectively. The cell proliferation was assessed by confluence measurements normalized to 0 h calculated using IncuCyte (Essen BioScience). The experiments were performed in triplicate.

To gain the more comprehensive cytotoxic information upon CQ and HCQ treatment, we chose eight different types of cell lines, which included IMR-90, A549, ARPE-19, Hep3B, Vero, HEK293, H9C2, and IEC-6. This panel includes the normal diploid cells and transformed and tumor cell lines which can represent different originated tissue to some extent. To evaluate the cytotoxicity of CQ we used the long-term dynamic cell image acquisition device Incucyte S3, which can take photos of cells in each group every 3 h. Then the confluence of each group was measured and analyzed by these photos compared with control group. The pictures reflecting the effects of CQ and HCQ on cell morphology and confluences are shown in Supplementary Figure S2. Results from *in vitro* cytotoxicity study showed that CQ exhibited significant cytotoxic effects at 48 h when the dosing regimen was more than 30 μM. CQ was found to decrease the cell proliferation in a dose-dependent manner. When the concentration of CQ was more than 300 μM, most of the eight cell lines showed immediate toxicity within 3 h (Figure 1). Data previously reported showed that HCQ also has good antiviral activity for both treatment and pretreatment choice against SARS-CoV-2 (Yao et al., 2020). Similar to assessment of CQ toxicity, we also tested the effect of HCQ on the viability and proliferation of eight cell lines. The results showed that HCQ exhibited significant cytotoxicity at 48 h when the dosing regimen was more than 100 μM. HCQ inhibited the viability of most of these cells in a dose- and time-dependent manner. Among the eight cell lines, H9C2, HEK293, and IEC-6 are the most sensitive cell lines to HCQ based on the CC50–48 h (Figure 2).

### CC50 of Chloroquine and Hydroxychloroquine

In this study, CC50 values (half cytotoxicity concentration) for CQ and HCQ were measured at 48 and 72 h, respectively (Table 1; Supplementary Figure S3). As shown in Figures 1 and 2, when the concentration of CQ or HCQ is higher than 300 μM, the proliferation shows a sudden decline or brake compared with lower dosing regimens. H9C2, HEK293, and IEC-6 are the more sensitive cells to CQ compared with five other cell lines, as their CC50 value at 72 h is less than 20 μM (17.1, 9.883, and 17.38 μM, respectively). Additionally, the CQ exhibits mild cytotoxic activity on Vero and ARPE-19 cell lines with CC50 values of 92.35 and 49.24 μM at 72 h, respectively. Similar with CQ, HCQ exhibits strong cytotoxicity on H9C2, HEK293, and IEC-6 with CC50 values at 72 h lower than 30 μM (25.75, 15.26, and 20.31 μM at 72 h, respectively). HCQ exhibits weak cytotoxic activity on Vero and ARPE-19 cell lines with CC50 values of 56.19 μM and 72.87 μM at 72 h, respectively. Furthermore, the results of S9-mix assay demonstrated that the toxicity is mainly contributed by CQ and HCQ instead of their metabolites (Supplementary Figure S4).

**TABLE 1:** The CC50 of CQ and HCQ in different types of cell lines.

Cell lines	Tissue type	Drugs	21% O2		5% O2		1% O2	
			CC50–48 h	CC50–72 h	CC50–48 h	CC50–72 h	CC50–48 h	CC50–72 h
			(μM)	(μM)	(μM)	(μM)	(μM)	(μM)
H9C2	Heart	CQ	41.62	17.10	40.35	16.31	30.72	16.19
		HCQ	58.17	25.75	37.27	16.91	33.41	17.88
Hep3B	Liver	CQ	130.8	81.13	91.72	43.81	95.51	38.77
		HCQ	101.0	69.56	85.34	44.94	59.08	45.46
HEK293	Kidney	CQ	19.80	9.883	18.63	10.65	14.5	14.33
		HCQ	55.95	15.26	16.19	9.965	12.88	13.37
Vero	Kidney	CQ	48.61	92.35	N/A	N/A	N/A	N/A
		HCQ	58.22	56.19	N/A	N/A	N/A	N/A
IMR-90	Lung	CQ	63.24	35.43	96.53	61.5	10.99	9.675
		HCQ	56.02	45.24	89.98	74.18	18.25	14.62
A549	Lung	CQ	46.00	24.63	N/A	N/A	N/A	N/A
		HCQ	59.86	33.05	N/A	N/A	N/A	N/A
ARPE-19	Retina	CQ	195.4	49.24	N/A	N/A	N/A	N/A
		HCQ	208.3	72.87	N/A	N/A	N/A	N/A
IEC-6	Intestine	CQ	19.35	17.38	N/A	N/A	N/A	N/A
		HCQ	22.14	20.31	N/A	N/A	N/A	N/A

The CC50 on 24, 48, 72 h of CQ and HCQ decreased in a time-dependent manner, which suggests the cumulative toxic effect in most of the eight cell lines except Vero. As shown in Table 1, the CC50 value of 72 h increased instead of decreasing compared with that of 48 h in Vero, which may be due to special drug metabolism or stability in it.

### The Effect of Chloroquine and Hydroxychloroquine on Cell Cycle and Apoptosis

**FIGURE 3:**
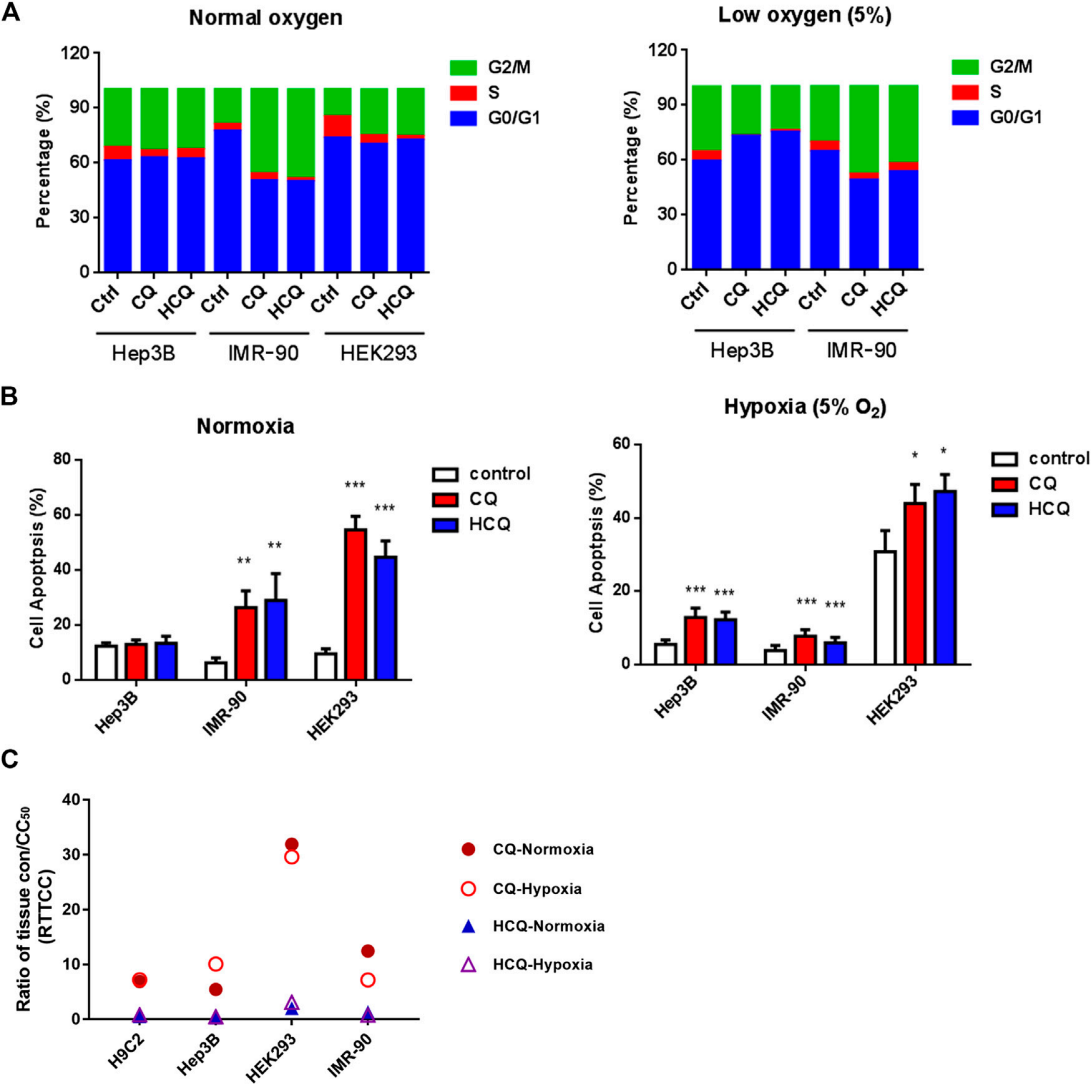
The effect of CQ and HCQ on cell cycle, apoptosis, and the predicted risk of cytotoxicity by PBPK model. **(A)** CQ and HCQ (50 μM) induced cell cycle arrest were analyzed by FACS at normoxia or hypoxia condition. **(B)** CQ and HCQ (50 μM) induced cell apoptosis were analyzed by FACS at normoxia condition and hypoxia analyzed by FACS. *, *p* < 0.05, **, *p* < 0.01, ***, *p* < 0.001. **(C)** Analysis of ratio of tissue trough concentration vs. CC50 (RTTCC) in four cells under normoxia or hypoxia based on CQ and HCQ tissue concentration simulated by the physiologically based pharmacokinetic (PBPK) model by blood data after intravenous administration.

In addition to studying the effects of the two drugs on cell proliferation, we also analyzed the effects of CQ and HCQ on cell cycle and apoptosis. Our results showed that both CQ and HCQ could lead to S phase reduction significantly (Figure 3A). Upon hypoxia treatment, CQ and HCQ induce obvious arrest of HEK293 and Hep3B, which is more serious in HEK293. Moreover, both CQ and HCQ induced significant apoptosis of HEK293 in normoxia and hypoxia (Figure 3B). But the effect is a little weak on IMR-90 and Hep3B. Compared to IMR-90, HEK293 and Hep 3B are more sensitive to hypoxia condition.

### The Effect of Hypoxia on Toxicity of Chloroquine and Hydroxychloroquine

To simulate the hypoxia of pneumonia in COVID-19 patients and other diseases, we also test the cytotoxicity of CQ and HCQ under 5 and 1% oxygen concentration in parallel with normal concentration of oxygen, respectively. Cytotoxicity tests were carried out in H9C2, Hep3B, IMR-90, and HEK293, which represents four important organs, heart, liver, lung, and kidney, and the results are shown in Table 1. Under 5% oxygen concentration, hypoxia could increase the toxicity of HCQ in H9C2, Hep3B, and HEK293, but not in IMR-90, and increase the toxicity of CQ in Hep3B. The CC50 at 1% oxygen concentration decreased both in CQ and in HCQ which indicates hypoxia can enhance the cytotoxicity to some extent. To clarify the effects of hypoxia on cell viability, we compare normoxia with 5 and 1% oxygen condition. The results showed that H9C2, Hep3B, and IMR-90 are not sensitive to hypoxia (Supplementary Figure S5). However, hypoxia has a strong effect on HEK293 cells, which seriously affects the viability of 293 cells (Figure 3B and Supplementary Figure S5).

### PBPK Model and Risk of Toxicity

Using our PBPK models, we simulated the tissues concentrations of HCQ (600 mg BID for 1 day, 200 mg BID for days 2–5) and CQ (500 mg BID for 7 days) (Adelusi and Salako, 1982; Chhonker et al., 2018). The Cmax of tissue concentrations was summarized in Supplementary Table S1. Results of simulated tissue concentration showed that tissue trough concentration of CQ in liver and lung reached the highest level of drug accumulation (227.545 μg/ml), which is three times more than that in heart (60.598 μg /ml). However, the tissue trough concentration of HCQ in lung is the highest level (25.633 μg/ml) compared with liver, kidney, and heart (Supplementary Table S1 and Figure 3C).

In order to better predict the toxicity risk of CQ and HCQ in different tissues, we used the ratio of simulated tissue trough concentration to CC50 (R_TTCC_) to predict the risk of tissue toxicity for the safety profile of these two drugs in the given tissues. As shown in Figure 3C and Supplementary Table S1, we systematically compared the toxicity between CQ and HCQ in hypoxia and normoxia. The R_TTCC_ value of CQ is 6–17 times more than that of HCQ in lung, heart, kidney, and liver in normoxia, and the R_TTCC_ value of CQ is 8–20 times more than that of HCQ in hypoxia, which suggests that the toxicity risk of HCQ in the above tissues is much lower than that of CQ.

## Discussion

CQ and HCQ, widely used as antimalarial and autoimmune diseases drugs, recently have been under such controversial conclusion in current COVID-19 pandemic. On one hand, it has been reported that both CQ and HCQ can be used for the treatment of COVID-19 infected patients, though the underlying mechanism is unclear (Vincent et al., 2005; Schrezenmeier and Dorner, 2020).

On the other hand, the latest clinical trials indicate the CQ/HCQ has no such effect on increasing negative conversion probability and reducing mortality (Magagnoli et al., 2020; Tang et al., 2020). On top of that, the severe side effect which includes the cardiopathy and retinopathy has hindered its further application and was suspended very lately. Nevertheless, repurposing of CQ or HCQ and reevaluating the safety as well as toxicity is still in the need of taking further investigation for fighting against COVID-19 emergency. Therefore, the potential toxicities of these medications, including gastrointestinal symptoms, cutaneous reactions, cardiotoxicity, hepatotoxicity, and in particular retinopathy, are urgent to pay special attention, especially for those elders with underlying diseases.

Our results from proliferation rate and morphology revealed that both CQ and HCQ have shown certain cytotoxicity in eight different types of cell lines in time- and dose-dependent manner *in vitro*, suggesting the necessity of short period administration clinically (Figures 1, 2; Supplementary Figures S2, S3). Moreover, the metabolites of CQ and HCQ (CQ or HCQ pretreated with S9 mix) showed only weak toxicity on these cells compared with CQ and HCQ (Supplementary Figure S4), which demonstrates that toxicity of CQ and HCQ mainly depends on the drug itself rather than its secondary metabolites. And both CQ and HCQ could induce cell cycle arrest and apoptosis which are consistent with the phenotype of proliferation and could partially explain the toxic effect of CQ and HCQ (Supplementary Figures 3A,B). Among these types of cell lines, it does show the different tolerant capacity manifested by varied CC_50_ value. For example, the most cytotoxic effect was found in HEK293 (embryonic kidney cell line) and IEC-6 (intestinal epithelial cells) treated by CQ or HCQ. Studies have shown that drug metabolism is significantly affected under hypoxia environment with changes of pharmacokinetics, expression, and function of drug-metabolizing enzymes and transporters (Min et al., 2019). We also detected the cytotoxicity upon hypoxia and found it was increased in Hep3B and H9C2 cells, indicating that the toxicity of CQ/HCQ in tissues such as the heart and liver may increase under hypoxemia (Table 1). Moreover, compared with other cell lines, hypoxia alone induces a significant reduction in the viability of HEK293 cells (Supplementary Figure S5).

Although the cytotoxicity was obtained by live cell imaging system *in vitro*, this cellular toxic response of CQ and HCQ may refer to the tissue toxicity or *vice versa* to some extent. To better investigate the potential toxicity *in vivo* and *in vitro*, we proposed R_TTCC_ (ratio of tissue concentration and CC_50_) derived from PBPK model to predict the risk of toxic profiles in different tissues. We compared the R_TTCC_ data collected from heart, liver, kidney, and lung and revealed HCQ has shown significantly safe profiles compared to that of CQ treatment. However, recent publication reported that CQ was safer than HCQ according to selectivity index (SI) (the ratio of the CC_50_ to EC_50_) (Liu et al., 2020; Yao et al., 2020). We speculate that the safety difference might be due to their complex pharmacokinetic characteristics *in vivo*, which possessed specific distribution and long half-life of around days. As a result, our data shows that kidney and lung are prone to the toxicity of CQ and HCQ (Figure 3C; Supplementary Table S1). In the meantime, considering the nonnegligible effect on cardiocytes and retina cells, most patients with severe symptoms are more likely to suffer the dysfunction in heart and eyesight with aging simultaneously. Therefore, ECG monitoring should be necessary during clinical usage, even for the patients only infected with COVID-19 but without the underlying diseases. In addition, more attention should be paid to the patients in the changes of their eyesight when using HCQ.

In this study, we perform dynamic imaging system to accurately and precisely monitor the whole proliferation process other than conventional CCK8 assay. Furthermore, R_TTCC_ value suggests that drug distribution should be taken into account in the assessment of its potential toxicity within the tissues. Although no agreements have been reached on the effectiveness of these candidate drugs in the prevention or treatment of COVID-19, our study may provide more details, new evaluating parameters, and deep insight into the safety profile of CQ and HCQ in further preclinical or clinical trials.

## Data Availability Statement

The raw data supporting the conclusions of this article will be made available by the authors, without undue reservation, to any qualified researcher.

## Author Contributions

JY, ZG, XL, MW, and YL performed the research. LX, DL, CS, and HL designed the research and contributed with analytical tools. JY, MW, XY, CC, QL, and ZG analyzed the data. JY, MW, LX, YL, and ZG wrote the manuscript. All authors contributed to the article and approved the submitted version.

## Funding

This work was supported by the MOST (Ministry of Science and Technology of the People’s Republic of China) Foundation for SARS-nCov-02 Research (grant No. 2020YFC0844500) and Bill & Melinda Gates Foundation [INV-015694].

## Conflict of Interest

The authors declare that the research was conducted in the absence of any commercial or financial relationships that could be construed as a potential conflict of interest.

## Glossary

SARS-CoV-2: severe acute respiratory syndrome coronavirus 2
ARDS: acute respiratory distress syndrome
CQ: chloroquine
HCQ: hydroxychloroquine
COVID-19: coronavirus disease 2019
SLE: systemic lupus erythematosus
EC50: concentration for 50% of maximal effect
CC50: the median cytotoxic concentration
PBPK: physiologically based pharmacokinetic models
R_TTCC_: ratio of tissue trough concentration vs. CC50
BID: bis in die
SI: selectivity index
Cmax: peak concentration
Fa: fraction absorbed
Ka: absorption rate constants
