# Supplementary material for: Cytotoxicity Evaluation of Chloroquine and Hydroxychloroquine in Multiple Cell Lines and Tissues by Dynamic Imaging System and Physiologically Based Pharmacokinetic Model
Source: Front Pharmacol. 2020 Nov 20;11:574720. doi: 10.3389/fphar.2020.574720 (PMC7919379; doi:10.3389/fphar.2020.574720)
Supplement: Supplementary file 1 [file presentation1.pptx]

## Slide 1
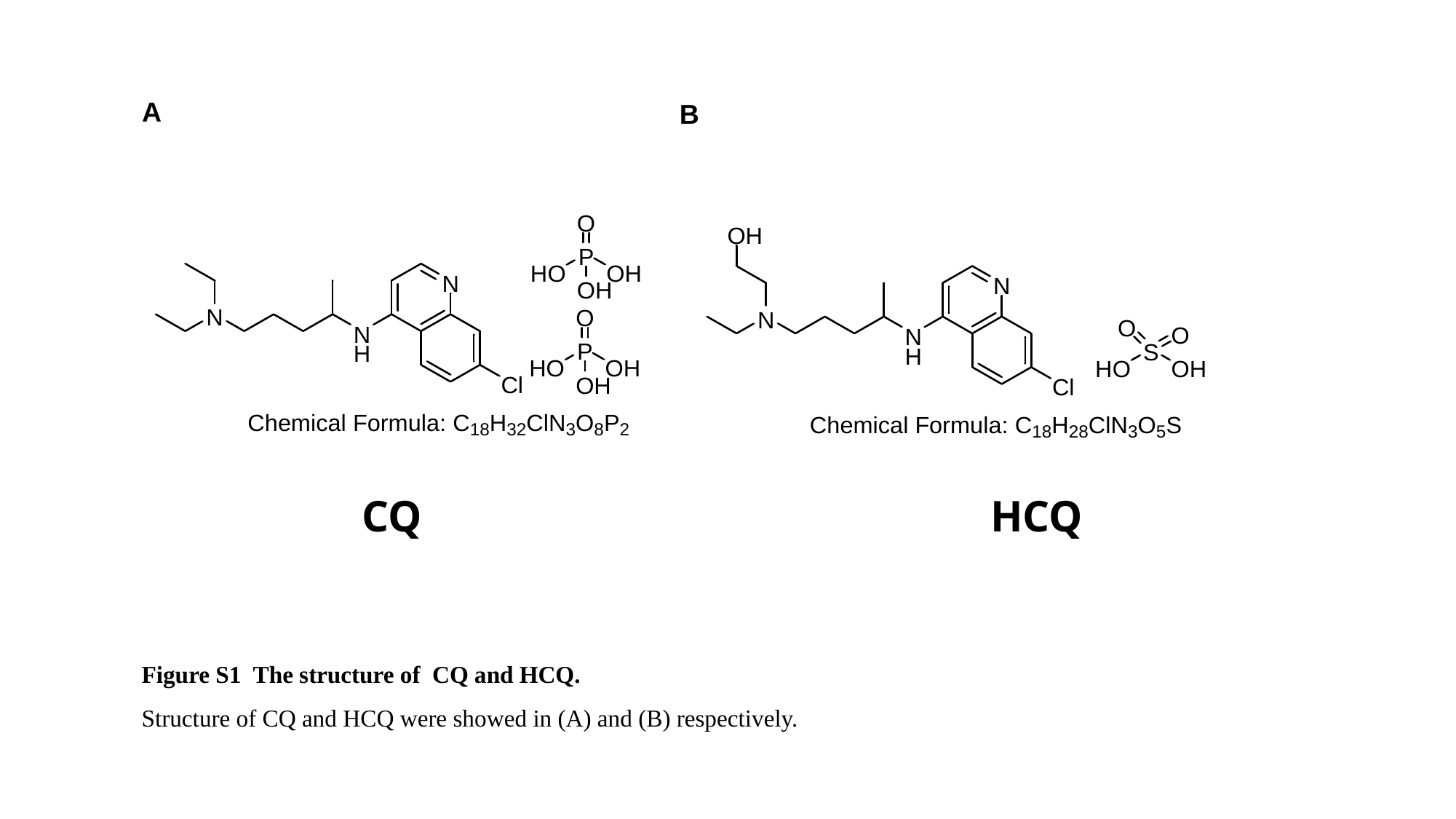

A
B
CQ
HCQ
Figure S1 The structure of CQ and HCQ.
Structure of CQ and HCQ were showed in (A) and (B) respectively.

## Slide 2
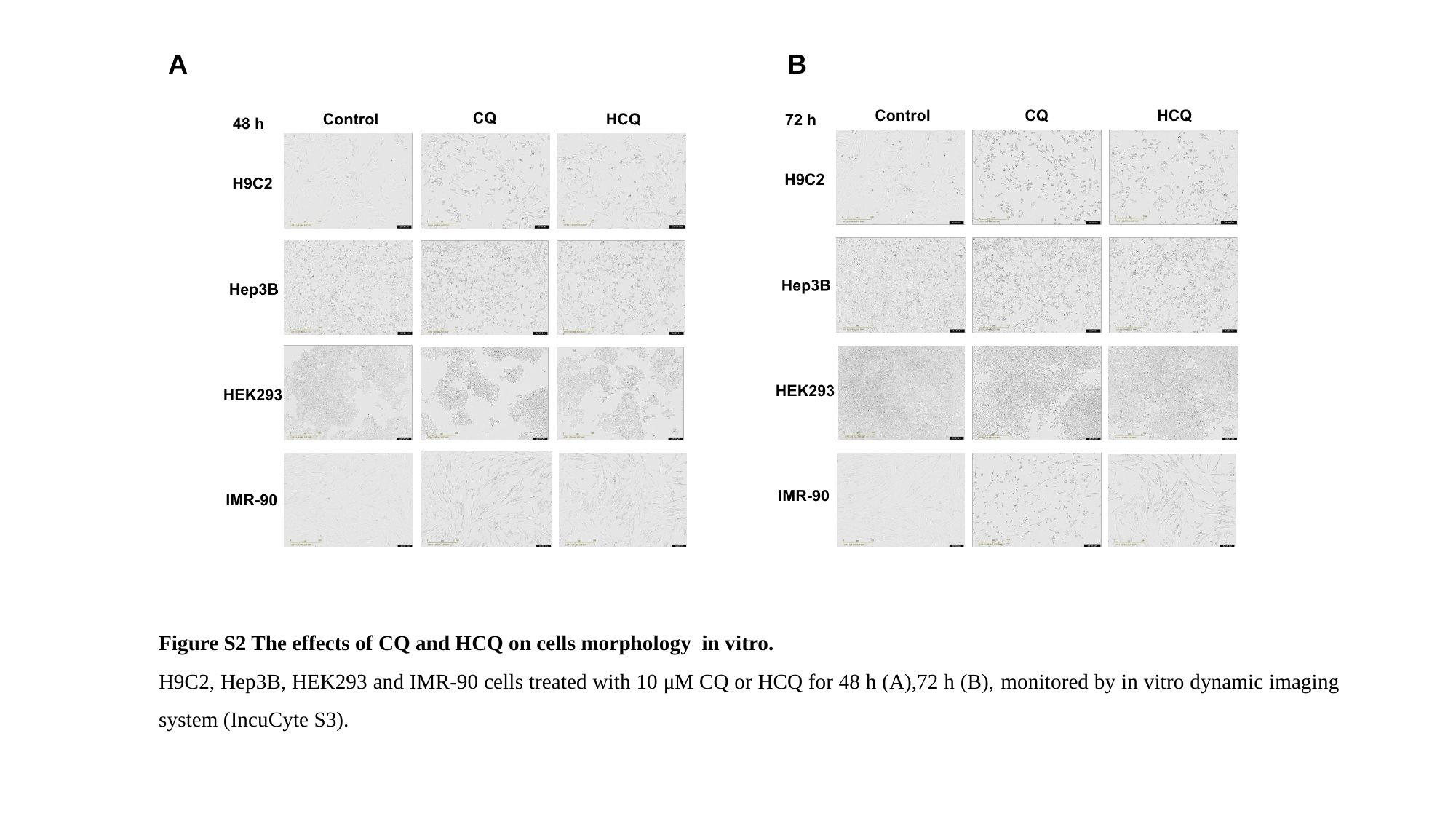

A
B
Figure S2 The effects of CQ and HCQ on cells morphology in vitro.
H9C2, Hep3B, HEK293 and IMR-90 cells treated with 10 μM CQ or HCQ for 48 h (A),72 h (B), monitored by in vitro dynamic imaging system (IncuCyte S3).

## Slide 3
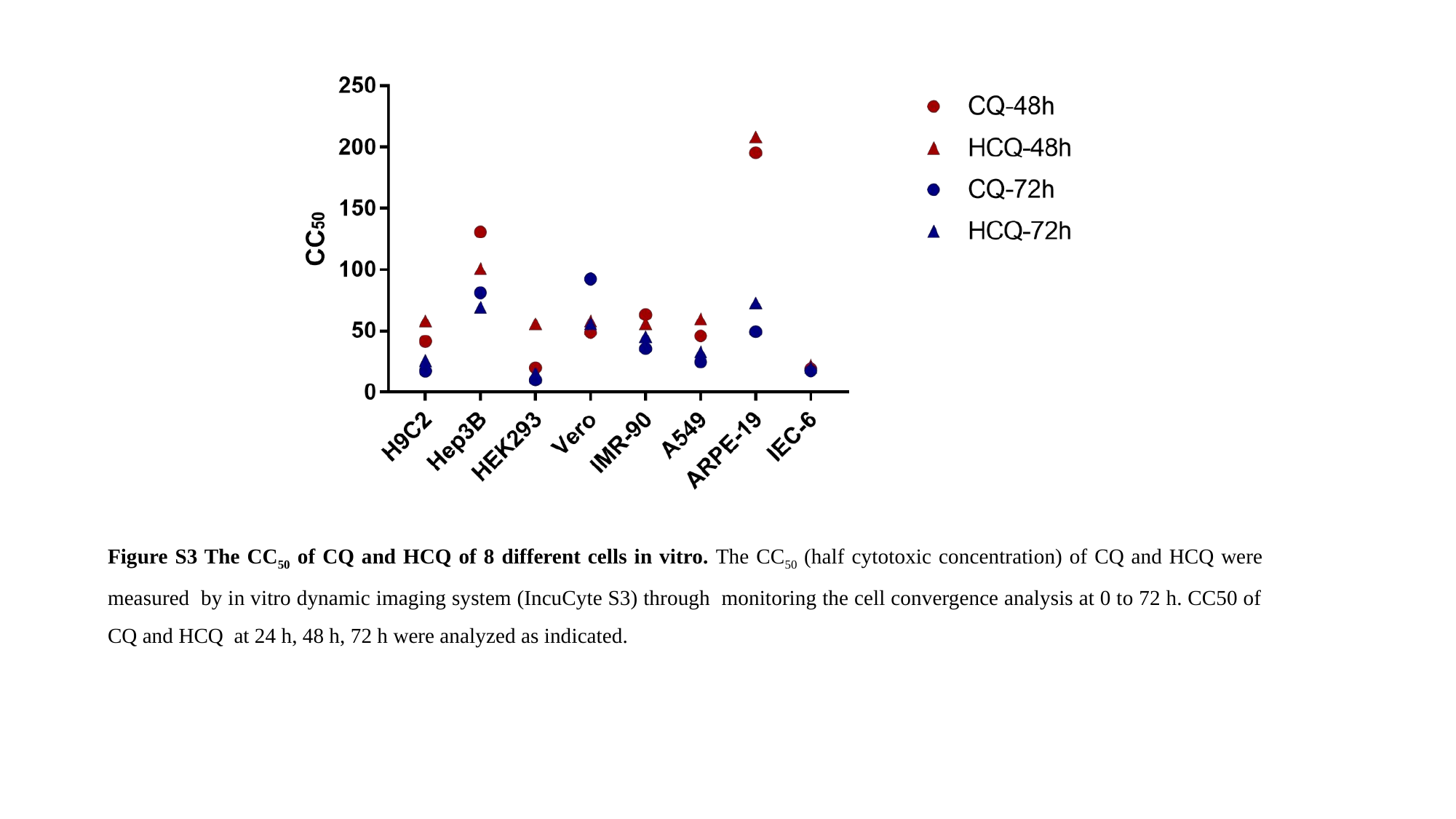

Figure S3 The CC50 of CQ and HCQ of 8 different cells in vitro. The CC50 (half cytotoxic concentration) of CQ and HCQ were measured by in vitro dynamic imaging system (IncuCyte S3) through monitoring the cell convergence analysis at 0 to 72 h. CC50 of CQ and HCQ at 24 h, 48 h, 72 h were analyzed as indicated.

## Slide 4
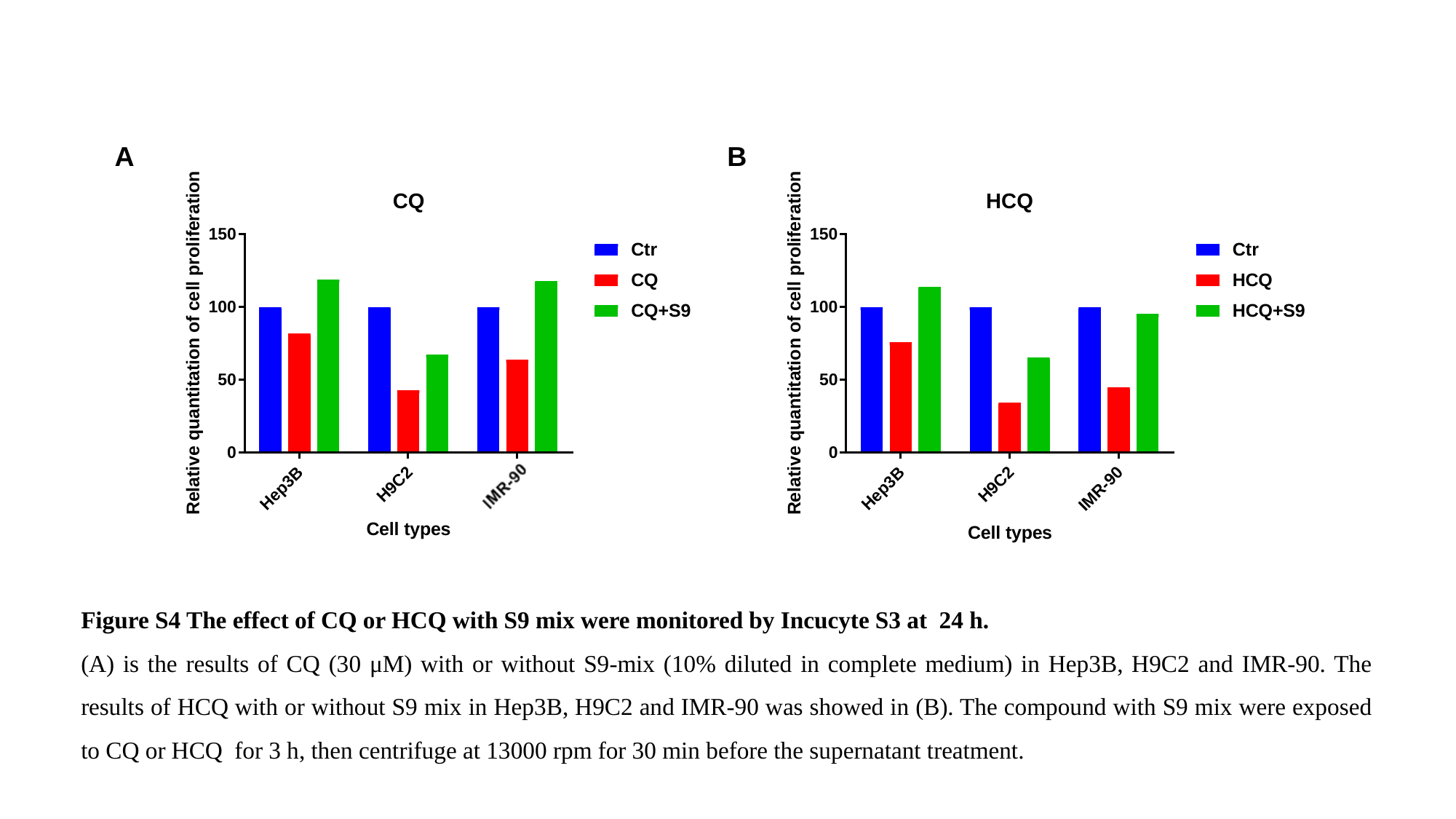

A
B
Figure S4 The effect of CQ or HCQ with S9 mix were monitored by Incucyte S3 at 24 h.
(A) is the results of CQ (30 μM) with or without S9-mix (10% diluted in complete medium) in Hep3B, H9C2 and IMR-90. The results of HCQ with or without S9 mix in Hep3B, H9C2 and IMR-90 was showed in (B). The compound with S9 mix were exposed to CQ or HCQ for 3 h, then centrifuge at 13000 rpm for 30 min before the supernatant treatment.

## Slide 5
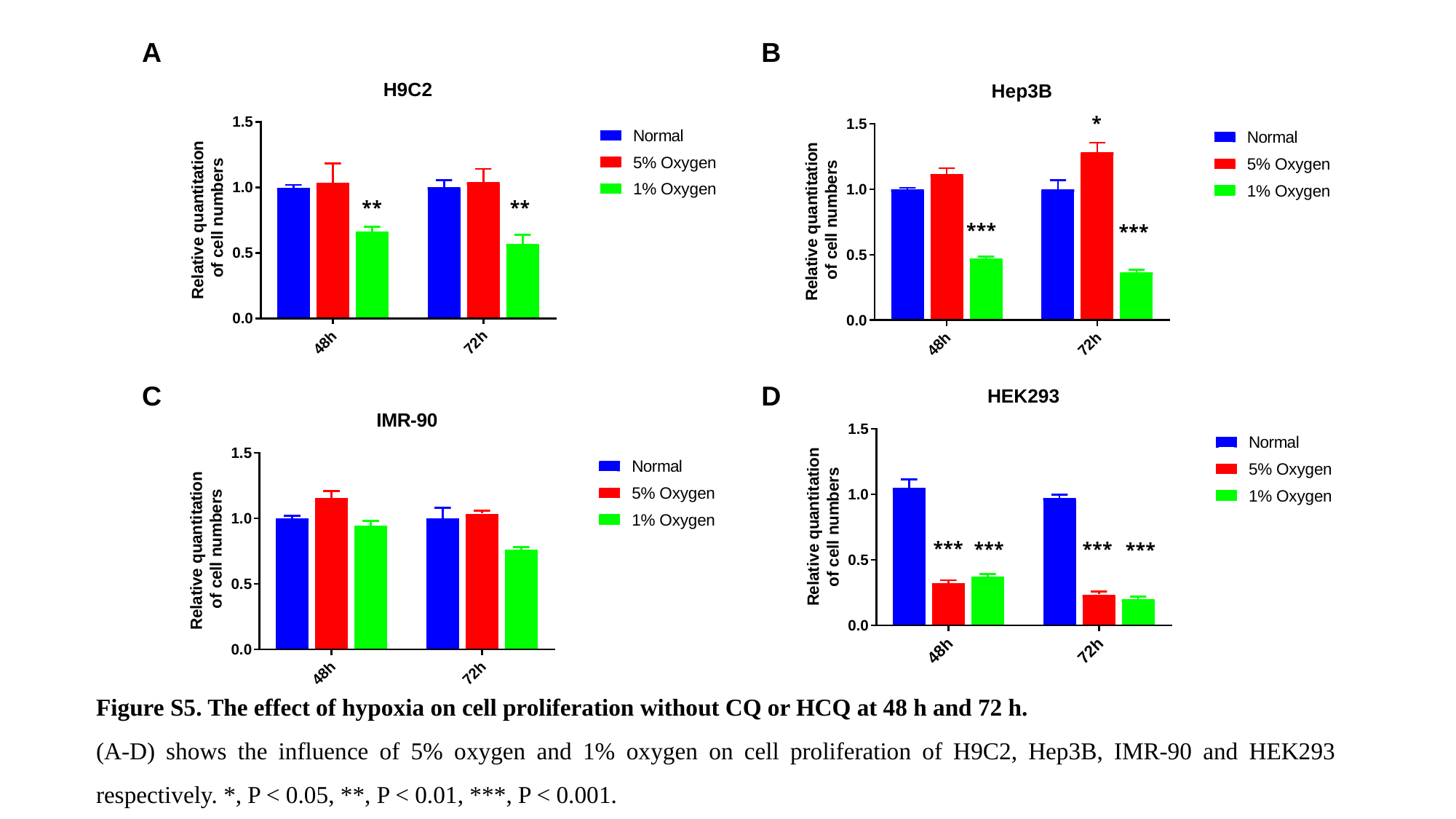

A
B
*
**
**
***
***
C
D
***
***
***
***
Figure S5. The effect of hypoxia on cell proliferation without CQ or HCQ at 48 h and 72 h.
(A-D) shows the influence of 5% oxygen and 1% oxygen on cell proliferation of H9C2, Hep3B, IMR-90 and HEK293 respectively. *, P < 0.05, **, P < 0.01, ***, P < 0.001.

## Slide 6
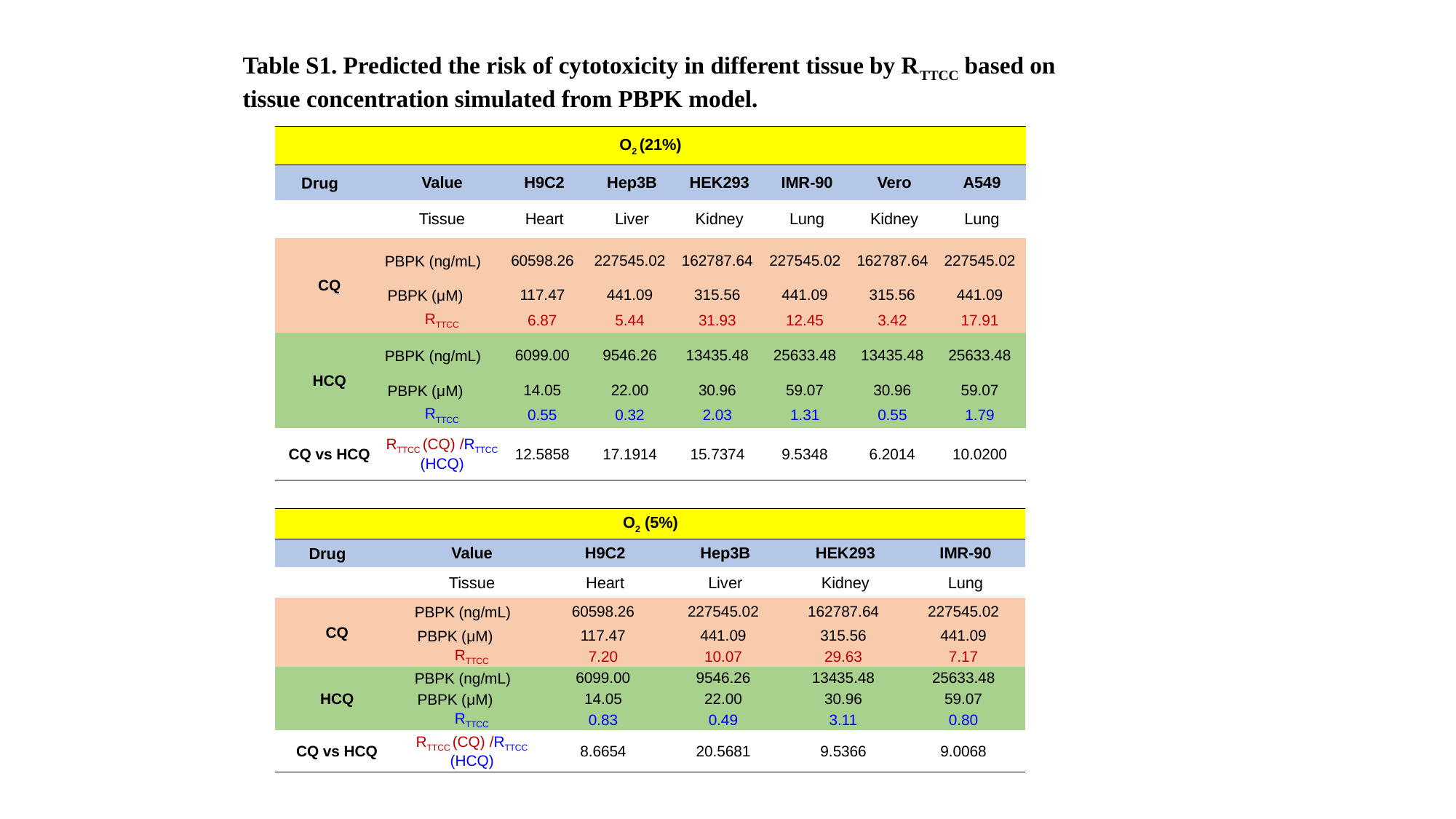

Table S1. Predicted the risk of cytotoxicity in different tissue by RTTCC based on tissue concentration simulated from PBPK model.
| O2 (21%) | | | | | | | |
| --- | --- | --- | --- | --- | --- | --- | --- |
| Drug | Value | H9C2 | Hep3B | HEK293 | IMR-90 | Vero | A549 |
| | Tissue | Heart | Liver | Kidney | Lung | Kidney | Lung |
| CQ | PBPK (ng/mL) | 60598.26 | 227545.02 | 162787.64 | 227545.02 | 162787.64 | 227545.02 |
| | PBPK (μM) | 117.47 | 441.09 | 315.56 | 441.09 | 315.56 | 441.09 |
| | RTTCC | 6.87 | 5.44 | 31.93 | 12.45 | 3.42 | 17.91 |
| HCQ | PBPK (ng/mL) | 6099.00 | 9546.26 | 13435.48 | 25633.48 | 13435.48 | 25633.48 |
| | PBPK (μM) | 14.05 | 22.00 | 30.96 | 59.07 | 30.96 | 59.07 |
| | RTTCC | 0.55 | 0.32 | 2.03 | 1.31 | 0.55 | 1.79 |
| CQ vs HCQ | RTTCC (CQ) /RTTCC (HCQ) | 12.5858 | 17.1914 | 15.7374 | 9.5348 | 6.2014 | 10.0200 |
| O2 (5%) | | | | | |
| --- | --- | --- | --- | --- | --- |
| Drug | Value | H9C2 | Hep3B | HEK293 | IMR-90 |
| | Tissue | Heart | Liver | Kidney | Lung |
| CQ | PBPK (ng/mL) | 60598.26 | 227545.02 | 162787.64 | 227545.02 |
| | PBPK (μM) | 117.47 | 441.09 | 315.56 | 441.09 |
| | RTTCC | 7.20 | 10.07 | 29.63 | 7.17 |
| HCQ | PBPK (ng/mL) | 6099.00 | 9546.26 | 13435.48 | 25633.48 |
| | PBPK (μM) | 14.05 | 22.00 | 30.96 | 59.07 |
| | RTTCC | 0.83 | 0.49 | 3.11 | 0.80 |
| CQ vs HCQ | RTTCC (CQ) /RTTCC (HCQ) | 8.6654 | 20.5681 | 9.5366 | 9.0068 |
